# Typhoid Fever without Abdominal Symptoms

**Published:** 1899-12-02

**Authors:** 


### TYPHOID FEVER WITHOUT ABDOMINAL SYMPTOMS.

At a recent meeting of the New York State Medical Association Dr. William Osler, speaking on the clinical diagnosis of typhoid fever, insisted on the frequency with which the disease runs its course without presenting its typical symptoms, and even without presenting any abdominal symptoms at all. This is a matter the due recognition of which is of very great importance, tending as it does on the one hand to narrow down the number of those vague cases which are commonly spoken of as simple continued fever, and on the other to offer a probable explanation of outbreaks occurring in places where no history of the introduction of the infection can be traced. By the use of the Widal test it has become pretty clear that many cases in which for from five to eight days there has been slight fever, and in some of which perhaps it has been possible to feel the edge of the spleen, have really been cases of typhoid fever, notwithstanding the absence of rose spots and of all the ordinary symptoms of that disease. Dr. Osler, indeed, says that out of 35 patients which had been under his care during the preceding month only four had had distinct abdominal symptoms, and he held that besides the enteric type there are forms of disease in which the cerebro-spinal, the pulmonary, and the renal symptoms are the most important. "Many cases of sporadic cerebro-spinal meningitis are undoubtedly typhoid fever of the meninges. . . . Undoubtedly certain cases of so-called acute nephritis are really renal typhoid." He goes on to say that there may be no fever at all; intestinal symptoms may be entirely absent, and there may be no diazo reaction; there may be no leucocytosis; and Widal's reaction may be positive only late in the disease, or may not occur until the fever has ceased. All this is, indeed, cutting ourselves adrift from our landmarks. Yet it is probably true, and certainly it is only in accord with what we have lately learned in regard to the byways of other infections.

